# The Effect of Risk-Reducing Salpingo-Oophorectomy on Breast Cancer Incidence and Histopathological Features in Women with a *BRCA1* or *BRCA2* Germline Pathogenic Variant

**DOI:** 10.3390/cancers15072095

**Published:** 2023-03-31

**Authors:** Annechien Stuursma, Bert van der Vegt, Liesbeth Jansen, Lieke P. V. Berger, Marian J. E. Mourits, Geertruida H. de Bock

**Affiliations:** 1 Department of Obstetrics and Gynecology, University Medical Center Groningen, University of Groningen, 9713 GZ Groningen, The Netherlands; 2 Department of Epidemiology, University Medical Center Groningen, University of Groningen, 9713 GZ Groningen, The Netherlands; 3 Department of Pathology & Medical Biology, University Medical Center Groningen, University of Groningen, 9713 GZ Groningen, The Netherlands; 4 Department of Surgical Oncology, University Medical Center Groningen, University of Groningen, 9713 GZ Groningen, The Netherlands; 5 Department of Clinical Genetics, University Medical Center Groningen, University of Groningen, 9713 GZ Groningen, The Netherlands

**Keywords:** *BRCA1/2*, breast cancer, RRSO/oophorectomy

## Abstract

**Simple Summary:**

Women with a *BRCA1/2* germline pathogenic variant (GPV) are advised to undergo surgery to remove their ovaries and fallopian tubes at a young age to prevent tubal/ovarian cancer. This surgery is called a risk-reducing salpingo-oophorectomy (RRSO). Previous studies have suggested that RRSO may also decrease breast cancer (BC) risk by decreasing female hormone levels. The aim of this prospective study was to investigate the effect of RRSO on the risk and histopathological features of BCs in these women. We linked data from our hospital-based data/biobank to data from the Dutch Nationwide Pathology databank (PALGA). We included 1312 women in our study with 164 diagnosed BCs. RRSO did not influence BC incidence and there were no differences in histopathological features between BCs before and after RRSO. Therefore, the purpose of RRSO remains to decrease tubal/ovarian cancer risk only.

**Abstract:**

Background: Risk-reducing salpingo-oophorectomy (RRSO) is advised for female *BRCA1/2* germline pathogenic variant (GPV) carriers to reduce tubal/ovarian cancer risk. RRSO may also affect breast cancer (BC) incidence. The aim was to investigate the effect of RRSO on BC incidence and histopathological features in female *BRCA1/2* GPV carriers. Methods: Prospectively collected clinical data from *BRCA1/2* GPV carriers in our hospital-based data/biobank were linked to the Dutch Nationwide Pathology Databank (PALGA) in January 2022. Multivariable Cox-proportional hazard models were used to calculate hazard ratios (HRs) with 95% confidence intervals (95% CIs), where the pre-RRSO group was considered the reference group and the primary endpoint was the first primary BC. Histopathological features of BCs pre- and post-RRSO were compared using descriptive statistics. Results: In 1312 women, 164 incident primary BCs were observed. RRSO did not decrease BC risk for *BRCA1* GPV (HR: 1.48, 95% CI: 0.91–2.39) or *BRCA2* GPV (HR: 0.95, 95% CI: 0.43–2.07) carriers. BCs tended to be smaller post-RRSO (median: 12 mm) than pre-RRSO (15 mm, *p*: 0.08). There were no statistically significant differences in histopathological features. Conclusions: RRSO did not decrease BC risk or affect BC features in *BRCA1/2* GPV in this study, although BCs diagnosed post-RRSO tended to be smaller.

## 1. Introduction

Women with a *BRCA1/2* germline pathogenic variant (GPV) have an increased risk to develop breast and ovarian cancer. The cumulative lifetime risk up to age 80 to develop breast cancer is 72% for *BRCA1* GPV (95% CI: 65–79%) and 69% for *BRCA2* GPV (95% CI: 61–77%). For tubal/ovarian cancer, the cumulative lifetime risk up to age 80 is 44% for *BRCA1* GPV (95% CI: 36–53%) and 17% for *BRCA2* GPV (95% CI: 11–25%) [1]. To decrease the risk of tubal/ovarian cancer, risk-reducing salpingo-oophorectomy (RRSO) is the only proven effective option. This reduces the risk of tubal/ovarian cancer by >96% if performed before the incidence rises (recommended age range: 35–40 years for *BRCA1* and 40–45 years for *BRCA2*) [2]. To reduce mortality and morbidity from breast cancer, early detection by breast cancer screening as well as risk-reducing mastectomy (RRM) are effective options [3]. 

It has been suggested that RRSO at premenopausal age also reduces the risk of breast cancer in *BRCA1/2* GPV carriers by up to 50% [4]. The hypothesis is that reduced exposure to ovarian hormones (estrogen and progesterone) may lead to a lower risk of developing breast cancer post-RRSO. However, in a series of 162 Dutch *BRCA1/2* GPV carriers who underwent RRSO with a median follow-up of 28 months, Fakkert et al. could not confirm this [5]. Additionally, a large proportion of studies reporting a risk-reducing effect of RRSO on breast cancer risk were subject to cancer-induced testing bias and immortal person-time bias [6,7,8,9]. After eliminating these types of bias, Heemskerk-Gerritsen et al. did not find breast cancer risk reduction post-RRSO (HR: 1.09, 95% CI: 0.67–1.77) in 822 Dutch *BRCA1/2* GPV carriers [10].

In a multi-center prospective cohort, Mavaddat et al. did not find a breast cancer risk-reducing effect of RRSO in 2272 women with a *BRCA1* GPV (HR: 1.23, 95% CI: 0.95–1.61) followed-up for a mean of 5.4 years [11]. For 1605 *BRCA2* GPV carriers, RRSO carried out before or after age 45 did not decrease breast cancer risk (HR_before 45 years_: 1.07, 95% CI: 0.69–1.64, HR_after age 45_: 0.68, 95% CI: 0.40–1.15), followed-up for a mean of 4.9 years. Nonetheless, HRs for *BRCA2* GPV carriers decreased with increasing time since RRSO (HR_5 years or longer after RRSO_: 0.51, 95% CI 0.26–0.99) [11]. Stjepanovic et al. suggested that the age at RRSO determines the breast cancer risk-reducing effect and that RRSO at a younger age would, therefore, be more protective because the exposure to ovarian hormones would be shorter [12]. Results from Choi et al. indicated that the effect of RRSO is temporary and mainly reduces breast cancer risk in the first years after RRSO [13]. To summarize, consensus on the effect of RRSO on breast cancer risk in *BRCA1/2* GPV has not yet been reached. 

Besides incidence, the histopathological features of the breast cancers that develop before and after RRSO may provide us with more insight into the effects of RRSO on breast cancer in *BRCA1/2* GPV carriers. In a case-control study, in which 20 primary breast cancers diagnosed post-RRSO were matched to 36 breast cancers of *BRCA1/2* GPV carriers pre-RRSO, Van Verschuer et al. showed that breast cancers post-RRSO were smaller (11 vs. 17 mm, *p* < 0.05), with lower mitotic activity index (12 vs. 22 mitotic counts/mm2, *p* < 0.05) [14]. They did not find a statistically significant difference in hormone and HER-2 receptor status. To the best of our knowledge, the abovementioned study was the first and only one to provide detailed data on this topic. Therefore, the primary aim of this study was to investigate the effect of RRSO in a large cohort of female *BRCA1/2* GPV carriers on breast cancer incidence and its histopathological features. We hypothesized that RRSO does not affect breast cancer incidence, but that tumors found post-RRSO are smaller and less aggressive compared to tumors diagnosed pre-RRSO. 

## 2. Materials and Methods

In January 2022 data from OncoLifeS, the hospital-based data- and biobank of the University Medical Center of Groningen (UMCG) [15] were linked to PALGA, the Dutch Nationwide Pathology Databank [16]. 

### 2.1. OncoLifeS 

In the UMCG, women with a hereditary risk to develop breast and/or tubal/ovarian cancer are referred to the family cancer clinic for counseling on the choice and timing of genetic testing and possible risk-reducing strategies to prevent (mortality from) breast and tubal/ovarian cancer [16]. 

Since 2009, ovarian cancer screening is no longer advised for women with a *BRCA1/2* GPV in our hospital [17,18]. Instead, all women are advised to undergo timely RRSO after completion of childbearing between the ages of 35–40 years in case of a *BRCA1* GPV and 40–45 years for a *BRCA2* GPV. If DNA testing is performed before reaching the recommended age range, uptake of RRSO during the recommended age range is high (95%) in our cohort [19]. 

From 1994, after written informed consent, all newly referred women to the family cancer clinic are prospectively included in our data/biobank, OncoLifeS [15]. In OncoLifeS, routine clinical data are linked to questionnaires and biological specimens. OncoLifeS has been approved by the medical ethics committee of the UMCG and was registered in the Dutch Trial Register under the number: NL7839. In the OncoLifeS consent form, permission is asked to link to other data sources.

### 2.2. PALGA 

Pathology excerpts were requested by the research board of PALGA. Data available from PALGA are, amongst others, tumor type, tumor size, grade, metastatic regions, lymph node involvement, hormone and HER2 receptor status and surgical margins, if applicable. Pathology reports from all diagnosed breast cancers, tubal/ovarian cancers, RRMs and RRSOs were requested for this study. PALGA has had nationwide coverage since 1 January 1989 [16].

### 2.3. Data Collection 

Clinical data retrieved from OncoLifeS were the date of birth, mutation type, status and date of DNA test, status and date of RRM, status and date of RRSO and date and type of breast cancer surgery. A link with the Dutch Personal Records Database was performed for all participants to retrieve the date of death. The following breast cancer features were collected from PALGA: date of diagnosis, tumor type, size, T-stage, N-stage and hormone- and HER2-receptor status. From PALGA excerpts, date and histopathological features of tissue from RRMs and RRSOs could also be retrieved. All PALGA excerpts were thoroughly reviewed (AS) and in case of uncertainties the report was checked by our pathologist (BV). All secondary cancers were compared to the primary cancers and reviewed together with our pathologist (BvdV) to determine if it was a local recurrence or a second primary tumor. 

### 2.4. Study Population and Observation Period

Women were selected from OncoLifeS if they had a proven *BRCA1* or *BRCA2* GPV and were 25 years or older at the time of data linkage (January 2022), since the Dutch guidelines recommend yearly screening for women with a *BRCA1/2* GPV from age 25 onwards [20]. The observation started at age 25 or the date of the DNA test if the DNA test was performed after age 25. The observation ended on the date of the first primary breast cancer diagnosis, date of ovarian cancer diagnosis, date of RRM, date of death or date of PALGA linkage (January 2022), whichever came first.

### 2.5. Statistical Analyses 

Descriptive statistics were performed to describe the patients in the pre-RRSO group versus the post-RRSO group. Prevalent and incident breast cancers diagnosed pre- and post-RRSO were described. Prevalent cancers were considered all primary breast cancers that were diagnosed in this cohort before the DNA-test date or before age 25 (before the start of breast cancer screening). Breast cancers were considered incident cancers if they were diagnosed after the DNA-test date of the individual and after age 25. Tumor size, T-stage, N-stage and hormone- and HER2-receptor status were only shown for invasive cancers. 

The breast cancer incidence rate per 1000 person-years was calculated for both *BRCA1* and *BRCA2* GPV, stratified per pre-RRSO and post-RRSO groups. Person-years pre-RRSO were attributed to the pre-RRSO group, including a 6-month latency period post-RRSO, i.e., breast cancers diagnosed within 6 months post-RRSO were attributed to the pre-RRSO group. After this latency period, which was based on *BRCA*-specific tumor-volume doubling time [21], person years were attributed to the post-RRSO group. 

Multivariable Cox-proportional hazard models were used to assess the effect of RRSO on breast cancer risk, the primary endpoint being the first diagnosis of breast cancer. Hazard ratios with 95% confidence intervals (95% CIs) were calculated, where the pre-RRSO group was considered the reference group. Separate analyses were performed for *BRCA1* and *BRCA2* GPV. All models were corrected for age at baseline and birth year. RRSO was coded as a time-dependent variable, including a latency period of 6 months post-RRSO. A sensitivity analysis was performed in which the effect of undergoing RRSO before age 45 was assessed (within the recommended age range for *BRCA2*), where RRSO was coded as a time-dependent variable and women who underwent RRSO after age 45 were attributed to the pre-RRSO group. 

To compare breast cancer characteristics pre- and post-RRSO, Chi-square tests were performed for categorical data. For non-parametric data, Mann–Whitney U tests were performed. IBM SPSS statistics package version 28 was used for all analyses. *p*-values were considered significant if *p* < 0.05. 

## 3. Results

### 3.1. Study Population 

In Table 1, characteristics of the 1312 women with a *BRCA1/2* GPV included in this study are shown, of which 725 had a *BRCA1* GPV and 587 had a *BRCA2* GPV. The median observation time after the date of the DNA-test was 5.3 years (IQR: 1.5–9.8). A number of 798 women had undergone RRSO at a median age of 43.9 years. Of the 502 breast cancers diagnosed, 338 breast cancers were diagnosed before DNA-testing (prevalent cancers) and 164 were diagnosed after DNA-testing (incident cancers). A number of 373 women had undergone a censoring event before DNA-testing (28.4%), of which 23 (6.2%) had ovarian cancer and 15 (4.0%) had undergone bilateral mastectomy. In total 572 women (43.6%) underwent RRM and 82 (6.3%) were diagnosed with ovarian cancer. A number of 106 women (8.1%) developed a second primary breast cancer at a median age of 47.2 years, 8.5 years (median, IQR: 3.0–13.7) after the first diagnosis. Of those breast second primary breast cancers, 31 were diagnosed (6.0%) pre-RRSO and 7 (9.4%) post-RRSO. Of these breast cancers, 59 were in the contralateral breast. 

### 3.2. Breast Cancer Incidence Pre- and Post-RRSO

In Table 2, breast cancer incidence rates are shown for *BRCA1* and *BRCA2* GPV carriers, pre- and post-RRSO, per 1000 person-years of observation. In *BRCA1* GPV carriers, the incidence rate pre-RRSO was 34.4/1000 person-years, and post-RRSO 28.9/1000 person-years. In *BRCA2* GPV carriers, the incidence rate pre-RRSO was 25.7/1000 person-years and 20.5/1000 person-years post-RRSO. 

In Table 3, results from the Cox-proportional hazards model are shown, corrected for age at the start of observation and birth year. RRSO did not decrease breast cancer risk overall (HR: 1.23, 95% CI: 0.85–1.78), or for BRCA1 (HR 1.29: 95% CI: 0.81–2.05) or BRCA2 GPV separately (HR: 1.13, 95% CI: 0.62–2.06). In our sensitivity analysis, premenopausal RRSO < 45 years did not decrease breast cancer risk (HR: 1.31, 95% CI: 0.88–1.97).

### 3.3. Histopathological and Clinical Characteristics of Breast Cancers Pre- and Post-RRSO

In Table 4, incident cancers that were diagnosed after DNA tests of the individuals are shown. Overall, diagnosed breast cancers were most likely to be invasive (87.1%), grade III (62.0%) and staged T1 (74.8%). Sixty-one (45.9%) tumors were triple-negative breast cancers. Of the 164 breast cancers diagnosed, 103 were diagnosed pre-RRSO and 61 post-RRSO. Women with a first primary breast cancer diagnosed pre-RRSO were at a median age of 39.9 at the time of diagnosis and women with a primary breast cancer diagnosed post-RRSO were a median of 50.7 years at the time of diagnosis. Breast cancers diagnosed post-RRSO tended to be smaller (median 12 mm vs. 15 mm, *p* 0.08). 

Table S1 in the Supplementary Materials shows features of all breast cancers diagnosed in our population, including prevalent cancers, which were diagnosed before the DNA-test or before age 25. 502 breast cancers were retrieved from PALGA of which histopathological features were available in the excerpt for 498 cases. Pre-RRSO, 434 breast cancers were diagnosed and post-RRSO 65. Post-RRSO, tumors were of smaller size (median 12 vs. 19 mm, *p* < 0.001), more often staged T1 (79.4% vs. 53.9%, *p <* 0.05) and characterized as in situ (15.6% vs. 5.3%, *p* <0.05).

## 4. Discussion

The aim of this study was to investigate the effect of RRSO on breast cancer incidence and its histopathological features. In this cohort of 1312 women with a *BRCA1/2* GPV, after RRSO the breast cancer risk did not decrease for both *BRCA1* (HR: 1.29, 95% CI: 0.81–2.05) and *BRCA2* GPV carriers (HR: 1.13, 95% CI: 0.62–2.06) compared to pre-RRSO. There was no statistically significant difference in histopathological features between breast cancers diagnosed pre-RRSO and post-RRSO, although breast cancers diagnosed post-RRSO tended to be smaller (12 mm) compared to breast cancers diagnosed pre-RRSO (15 mm, *p* 0.08).

We did not find a risk-reducing effect of RRSO or RRSO <45 years on breast cancer incidence in our Cox-regression analysis, which corresponds with Heemskerk-Gerritsen et al. [10] and with Mavaddat et al. [11] (for *BRCA1* GPV carriers). Choi et al. [13] did find a risk-reducing effect of RRSO in a case-series of 2650 women with a *BRCA1* GPV (HR: 0.28, 95% CI, 0.10–0.63) and in 1925 women with a *BRCA2* GPV (HR: 0.19, 95% CI, 0.06–0.71), especially during the first 5 years post-RRSO. The crude-incidence rate of breast cancer post-RRSO was indeed lower in the current study, 25.0/1000 person-years post-RRSO vs. 30.5/1000 person-years pre-RRSO. As a direct result of the high uptake of RRSO after diagnosis of a *BRCA1/2* GPV in our cohort (95%) [19], the pre-RRSO group mainly contains person-years at risk of younger women (median year of birth 1978) and the post-RRSO group mainly of older women (median year of birth 1965). The breast cancer incidence between 25–40 years was previously observed at around 26% for *BRCA1* and 17% for *BRCA2* GPV carriers in our population by Van der Kolk et al., whereas between 40 and 55 years, a breast cancer incidence of 30% was observed in *BRCA1* and 42% for *BRCA2* GPV carriers [22]. If RRSO would not affect breast cancer incidence at all, one would have expected a somewhat higher risk of breast cancer in the post-RRSO group than found in the current analysis. 

In *Brca1*-knockout mice, breast tumor onset could statistically significantly be delayed after oophorectomy: median tumor onset was 300 days post-oophorectomy vs. 206 days without oophorectomy (HR 0.46, 95% CI 0.18–1.18, *p* 0.049) [23]. Another study in *Brca1*-mutant mice showed somewhat different results, namely that breast tumor incidence of oophorectomized and intact mice remains similar until 135 days post-oophorectomy. However, at 180 days, mammary tumor formation in oophorectomized mice was reduced by approximately 50%, with an average number of 1.1 tumors, compared to an average number of 1.7 tumors in non-oophorectomized mice [24]. Although we did not find a reduction in breast cancer risk post-RRSO, there still may be a (small) effect of RRSO on breast cancer growth. 

Our study results regarding the histopathological features of breast cancers after RRSO correspond with Verschuer et al. [14], who reported that tumors post-RRSO were smaller, with no statistically significant difference in tumor grade. Although breast cancers in *BRCA1/2* GPV carriers are more often triple-negative and have a higher histologic grade when diagnosed at a younger age [25,26,27], we did not find a difference in hormone receptor status or tumor grade pre- or post-RRSO. From mice studies, it became clear that *BRCA1*-associated tumorigenesis is influenced by progesterone-mediated activation of the RANKL/RANK/NF-κB pathway [28]. The *BRCA1*-associated tumors that develop are often ER- and PR-negative, but the possible ‘cell of origin’ in mice is RANK-positive, which is hyper-responsive to progesterone. Therefore, one might expect that premenopausal RRSO would decrease breast cancer risk because progesterone levels drop. In the Netherlands, however, the majority of women who have not been diagnosed with breast cancer are offered hormone replacement therapy (HRT) after premenopausal RRSO and women with a uterus in situ are offered progesterone-containing HRT [29]. This may have reduced the effect of RRSO on the BC risk in this population. 

To the best of our knowledge, this is the largest cohort in which histopathological features of breast cancers diagnosed pre- and post-RRSO are compared. A strength of this study is the use of PALGA data, which has excellent nationwide coverage and provided accurate histopathological information on RRMs, RRSOs, ovarian cancer and breast cancers in our cohort. Other strengths are the prospective inclusion of study participants and a relatively long median period of observation, 5.3 years. Furthermore, we avoided cancer-induced testing bias by starting observation after DNA-test of the individual and only including incident cancers, in addition to the avoidance of immortal person-time bias by treating RRSO as a time-dependent variable in our Cox-regression analysis. In the Netherlands, screening for breast cancer in this high-risk group begins at age 25. Therefore, breast cancers that occurred before the age of 25 years were not included in our analyses, because by definition, these are unscreened cancers, known to be larger [26,27]. This could otherwise have led to the biased interpretation that cancers detected before or without RRSO are larger and perhaps more aggressive. 

An important limitation of this study is that we could not correct for age in the comparison of histopathological features of breast cancers pre- and post-RRSO. Verschuer et al. [14] matched for age at diagnosis and found a smaller size of tumors diagnosed post-RRSO (11 mm) compared to tumors diagnosed pre-RRSO (17 mm, *p* 0.01), in addition to a lower mitotic count/mm2. Unfortunately, we did not have information on the mitotic activity index in the current study. Another limitation is our relatively small study population and a lack of information on important confounders, such as the use of hormone replacement therapy, smoking, parity, breastfeeding, and family history of breast cancer. 

Another possible explanation for not finding a risk-reducing effect of RRSO on breast cancer risk is that the optimal timing of RRSO to prevent tubal/ovarian cancer is between 35 and 40 years for *BRCA1* GPV carriers and between 40 and 45 years for *BRCA2* GPV. This may be too late to prevent hormone-related breast cancer in *BRCA1/2* GPV. In our cohort, the median age for primary breast cancer was 41.7 years in the pre-RRSO group, meaning that 50% of the women that developed breast cancer pre-RRSO, were diagnosed before the age of 42 years. However, considering the severity and burden of menopausal symptoms even 10 or more years post-RRSO [30], clinicians should be cautious in their communication regarding the possible protective effect of RRSO on breast cancer risk among women opting for it. The main reason to offer RRSO is and should be to reduce tubal/ovarian cancer risk.

## 5. Conclusions

In the current study, we did not find breast cancer risk reduction after RRSO in *BRCA1/2* GPV. Nonetheless, if RRSO would not affect breast cancer incidence at all, one would have expected a somewhat higher risk in the post-RRSO group. Breast cancers diagnosed post-RRSO seemed to be of smaller size compared to breast cancers diagnosed before RRSO. No statistically significant differences in the frequency of in situ carcinoma, breast cancer subtype, hormone and HER2 receptor expression or tumor grade were found. Larger studies with a longer observation time and more information on important confounders are necessary to draw final conclusions. The primary purpose of RRSO is still to prevent tubal/ovarian cancer. Information on the possible protective effect of RRSO on breast cancer risk should be conveyed cautiously to women opting for RRSO. 

## Figures and Tables

**Table 1:** Characteristics of women included in the study population.

Variables	Total Population (n = 1312)	Pre-RRSO (n = 514)	Post-RRSO (n = 798)
*BRCA* mutation, n (%)			
*BRCA1*	725 (55.3)	274 (53.3)	451 (56.5)
*BRCA2*	587 (44.7)	240 (46.7)	347 (43.5)
Year of birth, median (IQR)	1968 (1956–1978)	1978 (1957–1985)	1965 (1956–1972)
Age at RRSO, median (IQR)	NA	NA	43.9 (39.1–51.3)
Years of observation, mean (SD)	6.2 (5.1)	6.0 (4.7)	6.5 (5.3)
Median (IQR)	5.3 (1.5–9.8)	5.3 (1.8–8.7)	5.4 (1.4–10.4)
Age at DNA-test, mean (SD)	41.5 (13.8)	38.2 (17.1)	43.6 (10.7)
Median (IQR)	40.5 (31.0–50.3)	31.9 (25.5–50.0)	42.6 (36.3–50.5)
Censoring events, n (%)			
RRM	572 (43.6)	163 (31.7)	409 (51.3)
Ovarian cancer	82 (6.3)	62 (12.1)	20 (2.5)
Death	184 (14.0)	109 (21.2)	75 (9.4)
Event before DNA test	373 (28.4)	142 (27.6)	231 (28.9)
Age at censoring event, median (IQR)			
RRM	39.5 (32.8–46.5)	31.3 (27.7–35.6)	42.0 (36.8–47.9)
Ovarian cancer	52.0 (46.1–60.9)	51.7 (45.9–60.4)	53.1 (45.6–63.5)
Death	57.8 (48.4–70.4)	59.1 (47.1–71.2)	55.4 (50.0–69.3)
Primary breast cancer, n (%)	502 (38.3)	166 (32.3)	336 (42.2)
Age, median (IQR)	42.4 (36.2–50.6)	43.4 (34.3–53.0)	42.1 (36.6–50.0)

**Table 2:** Incidence rate of primary invasive breast cancers pre- and post-RRSO.

Variable	Cases	Person-Years at Risk	Incidence Rate per 1000 Person-Years of Observation
*BRCA1* and *BRCA2*			
All cases	164	5811	28.2
Pre-RRSO	103	3373	30.5
Post-RRSO	61	2438	25.0
*BRCA1*			
All cases	103	3175	32.4
Pre-RRSO	64	1858	34.4
Post-RRSO	38	1317	28.9
*BRCA2*			
All cases	61	2636	23.1
Pre-RRSO	39	1516	25.7
Post-RRSO	23	1120	20.5

**Table 3:** Risk of primary invasive breast cancer post-RRSO in *BRCA1/2* GPV carriers, adjusted for age at baseline and birth cohort.

Variable	Hazard Ratio (95% CI)	*p*-Value
*BRCA1* and *BRCA2*		
Pre-RRSO	1	
Post-RRSO	1.23 (0.85–1.78)	0.27
RRSO < 45 years	1.31 (0.88–1.97)	0.19
*BRCA1*		
Pre-RRSO	1	
Post-RRSO	1.29 (0.81–2.05)	0.28
RRSO < 45 years	1.48 (0.91–2.39)	0.11
*BRCA2*		
Pre-RRSO	1	
Post-RRSO	1.13 (0.62–2.06)	0.70
RRSO < 45 years	0.95 (0.43–2.07)	0.89

**Table 4:** Features of incident primary breast cancers, comparison pre- and post-RRSO (n = 164).

Features, n (%) (n Available)	All N (%) N = 164	Pre-RRSO N (%) N = 103	Post-RRSO, N (%) N = 61	*p*-Value Chi-Square or Mann-Whitney U
*BRCA1*	102 (62.2)	64 (62.1)	23 (37.7)	0.98
*BRCA2*	62 (37.8)	39 (37.9)	38 (62.3)	
Age at BC, median (IQR)	43.2 (36.2–52.4)	39.9 (35.5–51.7)	50.7 (45.4–55.5)	<0.001
Year of birth, median (IQR)	1968 (1956–1978)	1972 (1957–1979)	1964 (1956–1969)	0.002
Tumor type				0.76
In situ carcinomas	21 (12.9)	12 (11.8)	9 (14.8)	
Invasive (NST *)	138 (84.7)	87 (85.3)	51 (83.6)	
Lobular	4 (2.5)	3 (2.9)	1 (1.6)	
**Missing**	1	1	0	
Invasive tumor size				*0.08*
Median mm (IQR)	14 (9–22)	15 (10–22)	12 (8–17)	
Estrogen receptor status **				
Positive	65 (45.8)	41 (45.1)	24 (46.2)	0.99
Negative	77 (54.2)	50 (54.9)	28 (53.8)	
**Missing**	0	0	0	
Progesterone receptor status **				0.74
Positive	49 (34.8)	32 (35.2)	17 (33.3)	
Negative	93 (65.2)	59 (64.8)	35 (66.6)	
**Missing**	1	0	1	
Her-neu2 receptor status **				0.24
Positive	7 (5.3)	3 (3.5)	4 (8.7)	
Negative	133 (94.7)	82 (96.5)	43 (91.3)	
**Missing**	9	6	5	
Triple negative **	61 (45.9)	38 (44.7)	23 (48.9)	0.90
**Missing**	9	6	5	
Tumor grade **				0.73
I	6 (4.7)	3 (3.8)	3 (6.0)	
II	43 (33.3)	28 (35.4)	15 (30.0)	
III	80 (62.0)	48 (60.8)	32 (64.0)	
**Missing**	8	7	1	
Tumor stage **				0.48
pT1	95 (74.8)	57 (71.3)	38 (80.9)	
pT2	29 (22.8)	21 (26.3)	8 (17.0)	
pT3	3 (2.4)	2 (2.5)	1 (2.1)	
pT4	0	0	0	
**Missing**	11	7	4	
Lymph node status **				0.38
pN0	101 (74.8)	67 (77.0)	34 (70.8)	
pN1	29 (21.5)	16 (18.4)	13 (27.1)	
pN2	2 (1.5)	1 (1.1)	1 (2.1)	
pN3	3 (2.2)	3 (3.4)	0	
**Missing**	3	0	3	

* no special type. ** of invasive tumors.

## Data Availability

Data available on request due to ethical and privacy restrictions.

## Supplementary Materials

The following supporting information can be downloaded at: https://www.mdpi.com/article/10.3390/cancers15072095/s1, Table S1: Characteristics of primary breast cancers, including prevalent cancers, comparison pre- and post-RRSO (n = 498 breast cancers).
