# Supplementary material for: The Effect of Risk-Reducing Salpingo-Oophorectomy on Breast Cancer Incidence and Histopathological Features in Women with a BRCA1 or BRCA2 Germline Pathogenic Variant
Source: Cancers (Basel). 2023 Mar 31;15(7):2095. doi: 10.3390/cancers15072095 (PMC10093102; doi:10.3390/cancers15072095)
Supplement: Supplementary file 1 [file cancers-15-02095-s001.zip › cancers-2241228-supplementary.pdf]

Supplementary table

Table S1. Characteristics of primary breast cancers, including prevalent cancers, comparison pre- and post-RRSO (n=498 breast cancers)

**Characteristics (n available)**	**All, N (%) N=498**	**Pre-RRSO, N (%) N=434**	**Post-RRSO, N (%) N=65**	**P-value Chi-square or Mann-Whitney U**
BRCA mutation status				
BRCA1	291 (58.4)	250 (57.6)	41 (63.1)	
BRCA2	208 (41.8)	184 (42.4)	24 (36.9)	0.12
Age at BC, median (IQR)	42.4 (36.2-50.6)	41.7 (35.1-49.6)	49.1 (43.7-54.6)	<0.001
Year of birth, median (IQR)	1961 (1953-1970)	1961 (1952-1971)	1964 (1957-1969)	<0.001
Tumor type				0.015
In situ carcinomas	33 (6.6)	23 (5.3)	10 (15.6)	
Invasive NST*	445 (88.6)	393 (89.7)	52 (81.3)	
Lobular	2 (3.1)	17 (3.9)	2 (3.1)	
Missing	0	0	0	
Tumour size**				
Median mm (IQR)	18 (12-25)	19 (13-26)	12 (8-18)	<0.001
Missing	38	37	1	
Estrogen receptor status positive**	189 (48.1)	165 (48.5)	24 (45.3)	0.66
Missing	0	0	0	
Progesterone receptor status positive**	149 (38.5)	132 (39.4)	17 (32.7)	0.36
Missing	58	58	0	
Her-neu2 receptor status positive**	20 (6.4)	15 (5.6)	5 (10.4)	0.21
Missing	131	127	4	
Triple negative**	142 (40.2)	118 (39.1)	24 (47.1)	0.28
Missing	92	91	1	
Tumor grade**				0.29
I	15 (3.8)	12 (3.5)	3 (5.8)	
II	115 (29.1)	99 (28.9)	16 (30.8)	
III	265 (67.1)	232 (67.6)	33 (63.5)	
Missing	50	50	0	
Tumor stage**				0.006
pT1	238 (56.9)	199 (53.9)	39 (79.4)	
pT2	156 (37.3)	148 (40.1)	8 (16.3)	
pT3	174 (4.1)	15 (4.1)	2 4.1)	
pT4	7 (1.7)	7 (1.9)	0 (0.0)	
Missing	27	24	3	
Lymph node**				
pN0	299 (65.6)	263 (64.8)	36 (72.0)	0.39
pN1	111 (24.3)	98 (24.1)	13 (26.0)	
pN2	32 (7.0)	31 (7.6)	1 (2.0)	
pN3	13 (2.9)	13 (3.2)	0	
Missing	42	28	15	

* No special type

** Of invasive tumors
